# PANoptosis in cancer: bridging molecular mechanisms to therapeutic innovations

**DOI:** 10.1038/s41423-025-01329-z

**Published:** 2025-07-28

**Authors:** Jin-Fei Lin, Ting-Ting Wang, Ren-Ze Huang, Yue-Tao Tan, Dong-Liang Chen, Huai-Qiang Ju

**Affiliations:** 1 State Key Laboratory of Oncology in South China, Guangdong Provincial Clinical Research Center for Cancer, Sun Yat-sen University, Guangzhou, PR China; 2 Department of Clinical Laboratory, Sun Yat-Sen University Cancer Center, Guangzhou, PR China

**Keywords:** PANoptosis, Inflammatory programmed cell death, Tumor immune microenvironment, Immunotherapy, Cancer, Cell biology

## Abstract

PANoptosis, a newly defined inflammatory programmed cell death, plays key roles in tumor development and progression. This process involves the assembly of PANoptosome complexes under various stimuli, which activate multiple cell death pathways simultaneously. By integrating key sensors and effector molecules, PANoptosis enhances immunogenic cell death while counteracts immune evasion mechanisms. This review focuses on current research of PANoptosis in cancer. Clinically, PANoptosis-related signatures show clinical value for predicting patient survival, discerning tumor immune microenvironment (TIME) characteristics and evaluating the therapeutic response. Mechanistically, complex signaling networks regulate PANoptosis, which in turn influences tumor behavior through dynamic interactions with TIME components. Therapeutically, targeting PANoptosis-related pathways, including nanomedicine approaches, demonstrate encouraging preclinical results. Particularly, combining PANoptosis modulation with radiotherapy, chemotherapy, or immunotherapy enhances anti-tumor efficacy. These findings position PANoptosis as a promising therapeutic target for reshaping TIME, overcoming treatment resistance, and improving cancer outcomes. Future research will focus on elucidating context-dependent PANoptosome regulation and translating these insights into precision oncology strategies.

## Introduction

Dysregulated cell death, a fundamental hallmark of cancer, is closely associated with tumorigenesis and development [1]. The death modes, such as necrosis, apoptosis, and pyroptosis, can induce tumor cell death through different molecular mechanisms and signaling pathways to respond to exogenous environments and intracellular disorders [2, 3]. Inflammatory programmed cell death (PCD) refers to a regulated form of cell death that not only eliminates damaged, infected, or unnecessary cells but also actively triggers an inflammatory immune response, mainly including necroptosis, pyroptosis, and ferroptosis (an iron-dependent form of cellular demise instigated by lipid reactive oxygen species (ROS)) [4]. Besides, emerging evidence suggests that caspase-mediated apoptosis can also be inflammatory [4, 5]. Briefly, necroptosis, an lytic cell death pathway, is immunologically active, driven by receptor-interacting protein kinase 1/3 (RIPK1/3) and the effector mixed lineage kinase domain-like pseudokinase (MLKL) [6]. Pyroptosis is marked by inflammatory reactions that usually take place following the formation and stimulation of inflammasomes and executed by members of the gasdermin family [7, 8]. Apoptosis is a caspase-mediated pathway distinguished by the generation of apoptotic bodies, initiated by both internal and external cues [9]. Interestingly, cells carry out multiple PCD programs at the same time and exist extensive crosstalk under specific conditions, which is defined as PANoptosis.

PANoptosis, conceptualized by Kanneganti’s research team in 2019, refers to the phenomenon in which cells undergo pyroptosis, apoptosis and necroptosis simultaneously [10]. It acts as a unique inflammatory PCD pathway and a defensive response to external stimuli and pathogens, contributing to the maintenance of cellular homeostasis and overall stability, which was initially observed in macrophages responding to the influenza A virus [11]. It is worth noting that PANoptosis harbors the essential characteristics of above pathways, but cannot be explained by any one of them alone [12, 13]. Emerging evidence has established PANoptosis is governed by the assembly of PANoptosome complexes under various stimuli, which contains upstream receptors and molecular signals essential for activating multiple cell death pathways simultaneously, such as Z-DNA binding protein 1 (ZBP1)-PANoptosome and absent in melanoma 2 (AIM2)-PANoptosome.

The regulation of PANoptosis has been associated with various infections and inflammatory disorders [11, 14, 15]. Recently, investigations have revealed the significant role of PANoptosis in various malignancies [13, 16, 17]. PANoptosis-based molecular clustering and prognostic signature holds great potential in predicting patient survival and discerning tumor microenvironment (TME) traits [18, 19]. Targeting PANoptosis shows novel necessity in tumor treatment through multiple cell death pathways. For instance, in colorectal cancer, the increased cell death induced by cysteine desulfurase (NFS1) or Wilms tumor 1-associating protein (WTAP) depletion under oxaliplatin treatment cannot be completely reversed by any of the inhibitors of apoptosis, pyroptosis and necroptosis alone [13, 17]. PANoptosis triggers the release of a large number of inflammatory factors (such as IL-1β and IL-18), which significantly enhance anti-tumor immunity and can help reverse the immunosuppressive microenvironment typically induced by necroptosis alone [20, 21]. Wang et al. accurately localize tumor-specific PANoptosis activation using a “logic-gated” strategy, thereby avoiding the systemic inflammatory storm risk associated with targeted necroptosis induction [22]. Moreover, PANoptosis not only allows for the elimination of cancer cells by multiple cell death pathways, but also plays an important role in regulating the enrichment of effector or regulatory immune cells, thus participating in fine-tuning anti-tumor immunity within the TME and enhancing anti-tumor therapy effectiveness [23]. In addition, active investigation of the mechanisms and potential therapeutic agents, such as PANoptosis related agonists or inhibitors, that can regulate PANoptosis in cancer cells is likely to yield effective cancer treatments and improve patient outcomes [24]. Therefore, understanding the connection between PANoptosis and cancer may provide deeper insights into the occurrence and treatment of cancer, and may be critical in exploring new therapeutic strategies by interfering with the escape mechanism of PANoptosis in cancer cells. However, studies on these mechanisms are still in their infancy and require further exploration. This review discusses the regulatory mechanisms and prognostic role of PANoptosis in cancers. We focus primarily on the interplay of PANoptosis between cancer cells and the tumor immune microenvironment (TIME), and highlight the therapeutic implications of PANoptosis-targeting strategies in cancer therapy.

## From independent cell death pathways to PANoptosis as a “death continuum”

**Fig. 1:**
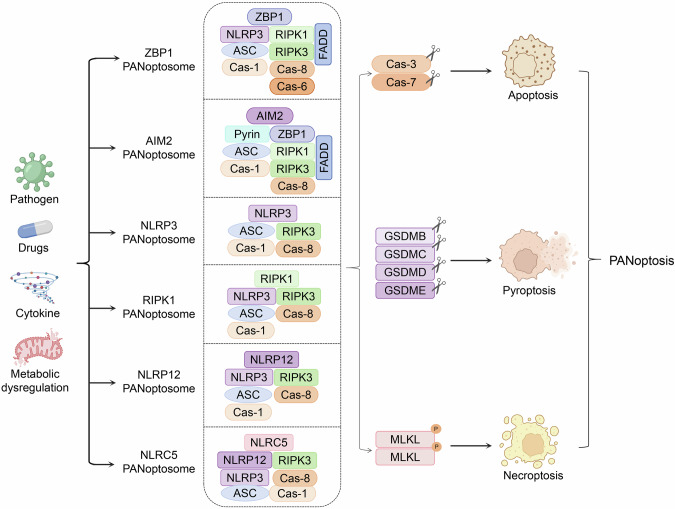
Mechanisms of PANoptosis activation. Under different extracellular or intracellular stimuli, such as pathogen infection, anti-tumor drugs treatment, cytokine storm and metabolic dysregulation, the ZBP1, AIM2, NLRP3, RIPK1, NLRP12 and NLRC5 sensor interact and recruit several adapter molecules to form PANoptosome, including ZBP1-PANoptosome (ZBP1, NLRP3, ASC, FADD, caspase-1/6/8 and RIPK1/3), AIM2-PANoptosome (AIM2, Pyrin, ZBP1, ASC, caspase-1/8, FADD and RIPK1/3), NLRP3-PANoptosome (NLRP3, ASC, RIPK3, caspase-1/8), RIPK1-PANoptosome (RIPK1/3, NLRP3, ASC, caspase-1/8), NLRP12-PANoptosome (NLRP12, NLRP3, ASC, caspase-1/8, and RIPK3), and NLRC5-PANoptosome (NLRC5, NLRP12, NLRP3, ASC, caspase-1/8 and RIPK3). These PANoptosomes further induce caspase-3/7 activation, GSDMB/C/D/E cleavage, and MLKL phosphorylation, resulting in apoptosis, pyroptosis, necroptosis

Each inflammatory PCD exhibit distinct morphological features, molecular mechanisms, and pathophysiological roles (Table 1). These processes are tightly regulated by upstream initiators and signaling cascades that assemble multimeric complexes to serve as activation platforms for downstream executioners. Given their critical role, these signaling molecules provide attractive target points for therapeutic intervention. Notably, emerging evidence highlights extensive crosstalk among these pathways under pathogenic or inflammatory stimuli, ultimately leading to PANoptosis [25]. The molecular machinery of PANoptosis revolves around the dynamic assembly of the PANoptosome, a multiprotein complex that integrates sensors, adapters, and effectors to synchronize pyroptosis, apoptosis, and necroptosis [26] (Table 2). The currently reported PANoptosomes include ZBP1-PANoptosome, AIM2-PANoptosome, NLRP3-PANoptosome, RIPK1-PANoptosome, NLRP12-PANoptosome, and NLRC5-PANoptosome (Fig. 1).

**Table 1:** Characteristics of the major types of inflammatory PCD

Features	Apoptosis	Pyroptosis	Necroptosis	Ferroptosis	PANoptosis
Definition	Classical PCD mediated by caspases, characterized by cell shrinkage and apoptotic bodies.	Inflammatory PCD activated by inflammasomes, leading to GSDMD/E cleavage and pore formation.	Regulated necrotic-like death mediated by RIPK1/RIPK3/MLKL signaling.	Iron-dependent lipid peroxidation driven	Composite PCD integrating pyroptosis, apoptosis, and necroptosis, regulated by PANoptosome
Initiators	Caspase-8/9, PARP1	Caspase-1/4/5/11/3/7	RIPK1, RIPK3	System x_c_-, GPX4, FSP1, ACSL4, TFR1	ZBP1, AIM2, RIPK1, NLRP12- PANoptosome
Executioners	Caspase-3/6/7, Bcl-2 family	GSDMB/C/D/E	MLKL	–	Caspase-3/6/7, GSDMB/C/D/E, MLKL
Morphological features	Cell shrinkage, DNA fragmentation, nuclear condensation, membrane blebbing, apoptotic body formation	Cell swelling, membrane blebbing, content release, pore and pyroptotic body formation	Increased membrane permeability and content release, organelles swelling, moderate chromatin condensation	Shrunken mitochondria with increased membrane density, loss of cristae	Multipathway features, combines pyroptotic swelling, apoptotic shrinkage, and necroptotic membrane rupture
Core regulatory mechanism	Caspase cascade, mitochondrial membrane permeabilization	Inflammasome activation, gasdermin protein cleavage	RIPK3-mediated MLKL phosphorylation and pore formation	Dysregulated iron metabolism, antioxidant system failure, lipid metabolic reprogramming	PANoptosome assembly and multipathway signal integration
Main triggers	DNA damage, growth factor withdrawal, death receptor activation	Infections (bacteria/viruses), metabolic stress (heme), endogenous damage (ATP, uric acid crystals)	Caspase-8 inhibition, viral infection	Iron overload, lipid peroxidation	Pathogen infection (viruses, fungi, bacterial), drugs, metabolic dysregulation, cytokine storms, chemotherapeutics, oxidative stress
Physiological function	Embryonic development, immune homeostasis, tissue renewal	Defense against intracellular pathogens	Antiviral immunity, tissue damage repair, antibacterial defense	Tumor suppression, immune regulations, cellular homeostasis	Tumor suppression, antiviral defense, inflammatory regulation, antibacterial immunity
Pathological significance	Cancer, neurodegenerative diseases, autoimmune diseases, ischemia/reperfusion injury	Cancer, sepsis, autoimmune diseases, metabolic diseases, cardiovascular diseases	Cancer, ischemia-reperfusion injury, neurodegenerative diseases, ischemia/reperfusion injury, autoimmune diseases	Cancer, tumor drug resistance, neurodegenerative diseases, liver disease, ischemia/reperfusion injury, infection, osteoarthritis	Infectious diseases, inflammatory diseases, cancer, sepsis, acute respiratory distress syndrome, and cytokine release syndromes, neurodegenerative disorders, cardiovascular diseases
References	[143–145]	[8, 56, 143, 144, 146]	[144, 147, 148]	[149–151]	[10, 23, 98, 99, 101, 152]

**Table 2:** Characteristics of PANoptosomes

PANoptosome type	Core sensor	Adapters	Effectors	Triggers	Disease contexts	References
ZBP1-PANoptosome	ZBP1, NLRP3	ASC, FADD	RIPK1/3, caspase-1/6/8	Viral RNA, DNA damage, IFNs + NEIs,	IAV infection, acute pancreatitis, spinal cord injury, cancer, sepsis	[28, 32, 33, 133, 153, 154]
AIM2-PANoptosome	AIM2, ZBP1, Pyrin	ASC, FADD	RIPK1/3, caspase-1/8	dsDNA viruses, bacterial infection	HSV-1 and *Francisella* infection, systemic lupus erythematosus, cancer	[14, 104, 106]
NLRP3-PANoptosome	NLRP3	ASC	RIPK3, caspase-1/8	ATP, crystals, K⁺/Ca^2+^/Na^+^ influx or mitochondrial ROS	Neurodegenerative disorders, cardiovascular diseases	[36, 38–42]
RIPK1-PANoptosome	RIPK1, NLRP3	ASC	RIPK3, caspase-1/8	TAK1 deficiency, TNF-α stimulation, Fungal and bacterial infection	*Yersinia* infection, asthma, acute liver failure, inflammatory bowel disease, ischemic brain injury	[44, 46, 47, 155, 156]
NLRP12-PANoptosome	NLRP12, NLRP3	ASC	RIPK3, caspase-1/8	TLR2/4 and IRF1 pathways, heme + DAMP/PAMP, malaria	Yersinia pestis or P. chabaudi infections, acute kidney injury, sickle cell disease	[50–53]
NLRC5-PANoptosome	NLRP12, NLRC5, NLRP3	ASC	RIPK3, caspase-1/8	TLR2/4 and IRF1 pathways, heme + DAMP/PAMP	Hemolytic disease, colitis, hemophagocytic lymphohistiocytosis	[28, 54, 157]

### ZBP1-PANoptosome

ZBP1 contains two Zα domains that recognize Z-form nucleic acids (Z-DNA/Z-RNA) and a RHIM domain mediating interactions with RIPKs [27]. ZBP1-PANoptosome was first identified during influenza A virus (IAV) infection, which includes core sensors ZBP1 and NOD-like receptor family pyrin domain-containing 3 (NLRP 3); adapters apoptosis-associated speck-like protein containing a CARD (ASC), and Fas-associated protein with a death domain (FADD) along with effectors caspase-1/6/8 and RIPK1/3 [28]. Caspase-6 can enhance complex stability by reinforcing RIPK3-ZBP1 interactions [14, 28, 29]. The ZBP1-PANoptosome coordinates apoptosis via caspase-3/7, necroptosis via MLKL, and pyroptosis via gasdermin D (GSDMD)/GSDME to eliminate infected cells [11]. The ZBP1-induced inflammatory pathway interacts with the cyclic GMP-AMP synthase (cGAS)- stimulator of IFN genes (STING) pathway, amplifying antiviral immunity [30]. Notably, genetic ablation of ZBP1 abolishes PANoptosis, impairing viral clearance [31], while inflammatory injury in acute pancreatitis and the pathological process of spinal cord injury can be significantly improved by inhibiting ZBP1- PANoptosome-mediated PANoptosis [32, 33].

### AIM2-PANoptosome

AIM2, an interferon-inducible cytosolic DNA sensor, plays a central role in antimicrobial defense. Its pyrin domain (PYD) domain binds ASC, while the HIN domain detects microbial dsDNA [14]. The AIM2-PANoptosome integrates signals from inflammasomes and recruits ZBP1, pyrin, ASC, FADD, caspase-1/8, and RIPK1/3 to drive pyroptosis, apoptosis, and necroptosis [14, 34, 35]. AIM2-knockout mice exhibit complete loss of PANoptosis during HSV-1 infection, leading to high mortality, whereas pyrin or ZBP1 deletion only partially reduces cell death, underscoring AIM2’s pivotal role [14]. AIM2-PANoptosome assembly depends on type I interferon (IFN-I) signaling, which upregulates ZBP1 and pyrin [14]. Single-cell RNA-seq data reveal that AIM2 deletion reduces ZBP1 expression in HSV-1-infected macrophages, indicating a feedback loop between AIM2 and ZBP1 [14]. Beyond infections, AIM2-PANoptosome contributes to autoimmune disorders. In systemic lupus erythematosus, AIM2 senses self-DNA released from neutrophil extracellular traps, promoting macrophage PANoptosis and type I interferon production [14]. This perpetuates autoantibody generation and organ damage.

### NLRP3-PANoptosome

NLRP3, the most versatile inflammasome sensor, is selectively expressed in myeloid cells and comprises three core domains: N-terminal PYD, a central NACHT domain with ATPase activity, and C-terminal leucine-rich repeats (LRRs) [36, 37]. Upon the activation by diverse stimuli (such as ATP, crystals, K⁺/Ca^2+^/Na^+^ influx or mitochondrial ROS), oligomeric complexes termed “specks” are formed in the cytoplasm, composed predominantly of ASC [38–40]. These specks recruit ASC, RIPK3, and caspase-1/8 complexes to form PANoptosome [41]. Beyond contributing to specific PANoptosomes, NLRP3 is integral to ZBP1-/RIPK1-/NLRP12-/NLRC5- PANoptosomes [42]. Several NLRP3 inhibitors are now in clinical trials for neurological and cardiovascular diseases, while some reported inhibitors exhibit poly-pharmacology, potentially affecting other cellular targets [36].

### RIPK1-PANoptosome

RIPK1 functions as a dual-function regulator, with its kinase activity initiating necroptosis and its scaffolding role facilitating apoptosis [43]. Under transforming growth factor beta-activated kinase 1 (TAK1) deficiency or TNF-α stimulation, RIPK1 orchestrates PANoptosome formation by recruiting NLRP3, ASC, RIPK3, and caspase-1/8 complexes [44]. TAK1 critically regulates this process, as both genetic ablation and pharmacological inhibition of TAK1 promote RIPK1-containing PANoptosome assembly [10]. However, subsequent investigations reveal that TAK1 ablation also activates RIPK1-independent PANoptosis through a distinct RIPK3-MLKL signaling axis, highlighting the existence of parallel regulatory mechanisms in inflammatory cell death programming [45]. Notably, RIPK1 deletion abolishes Yersinia-triggered pyroptosis and apoptosis but enhances ZBP1-mediated necroptosis, reflecting functional redundancy [44]. RIPK1 inhibitors are promising for treating inflammatory bowel disease, where RIPK1-PANoptosome activation drives epithelial cell death and barrier dysfunction [43, 46, 47]. In contrast, RIPK1 kinase-dead knock-in mice exhibit resistance to TNF-induced shock but increased susceptibility to bacterial infections, highlighting the delicate balance required for therapeutic intervention [48, 49].

### NLRP12-PANoptosome

NLRP12, a sensor with a leucine-rich repeat (LRR) domain, detects bacterial LPS, fungal components, and heme [50, 51]. It forms the NLRP12-PANoptosome with NLRP3, ASC, caspase-1/8, and RIPK3, driving PANoptosis during infections [50, 52]. TLR2/4 and IRF1 pathways regulate this complex, influencing intestinal immunity [50, 53]. NLRP12-PANoptosome activation correlates with acute kidney injury, and its knockout alleviates pathology [50]. In sickle cell disease, heme overload activates NLRP12-PANoptosome in renal tubular cells, exacerbating vaso-occlusive crises [50].

### NLRC5-PANoptosome

Recent studies have also revealed that NLRP12 can interact with NOD-like receptor family CARD domain-containing 5 (NLRC5), a critical innate immune sensor, inducing inflammatory cell death under hemolytic and inflammatory conditions [28]. The interaction between NLRC5 and NLRP12 facilitates the recruitment of NLRP3, ASC, RIPK3, caspase-1/8 to assemble the NLRC5-PANoptosome [54]. NLRC5 and NLRP12 exhibit distinct functional roles. A key distinction lies in NLRP12’s regulation of caspase-1 activation and inflammasome-dependent cytokine release, whereas NLRC5 does not influence these processes. Notably, the NLRP3-caspase-1 axis operates independently of NLRC5 [50, 54]). These findings highlight the subtle features of NLR family sensors within PANoptosome-mediated inflammatory cell death pathways.

PANoptosis involves extensive crosstalk regulated by PANoptosome complexes. For example, the activation of inflammasomes within the body, can activate the caspase protein family (such as caspase-1/4/5/11) or granzymes (such as granzyme A/B) to cleave and activate gasdermin proteins [8, 55]. As verified in apoptosis, caspase-3 can cleave the GSDME to potentially block pyroptosis via a different site from the inflammatory caspases that inactivate the protein [56, 57]. Caspase-8 is not only a key component of the exogenous apoptosis pathway, but also serves as a common molecular switch for apoptosis, necroptosis, and pyroptosis, functioning as a signaling hub for the overall mechanism [58]. Notably, RIPK1 deficiency abolishes pyroptosis and apoptosis induced by Yersinia but enhances ZBP1-mediated necroptosis [44]. Moreover, the same triggers can simultaneously activate multiple PANoptosomes, which is verified by the activated ZBP1- and AIM2-PANoptosome induced by viral infection (Table 2). In addition, activated ZBP1 promotes IFN-I secretion through the TANK binding kinase 1 (TBK1)–interferon regulatory factor 3 (IRF3) axis, thereby enhancing the formation of ZBP1- and AIM2-PANoptosomes and ultimately inducing PANoptosis [59]. Cytoplasmic mtDNA accumulation activates the AIM2 inflammasome, facilitating ZBP1-RIPK3 PANoptosome formation and inducing PANoptosis [60]. These highlight the crosstalk between the different cell death pathways. However, there is still much to learn regarding the regulators upstream of PANoptosome assembly and execution, and more PANoptosome complex may be discovered in the future.

## PANoptosis in cancer prognosis and TIME interplay

Cancer progression and treatment outcomes are the dynamic result of interaction and co-evolution between malignant cells and their surrounding TME, particularly within the TIME, whose main components include tumor cells, immune populations, various cytokines and inflammatory mediators [61, 62]. Recent research demonstrates the clinical relevance of PANoptosis patterns and its association with TIME in several types of cancers.

### The clinical relevance of PANoptosis-related signatures

PANoptosis exhibits key genetic and molecular features that regulate tumor progression [63]. Studies have reported that developing PANoptosis-related signatures (PANRS) through systematic analysis of PANoptosis-related genes (PRGs) or PANoptosis-related noncoding RNAs provides valuable clinical insights. These signatures enable effective prognostic stratification and precise assessment of therapeutic responses to anti-tumor therapy, such as chemotherapy or immunotherapy, highlighting the potential of PANRS to be used as an independent prognostic indicator.

Prognostic models and molecular subtyping systems based on PANRS demonstrate tumor-specific survival patterns across multiple malignancies. In prostate adenocarcinoma, comprehensive pathway analysis reveals interactions between PANoptosis-related genetic alterations (mutations, transcriptional dysregulation, methylation changes) and clinical features. Patients with elevated PANRS scores show improved survival, enhanced immunotherapy response, and elevated mutation burden [20]. Contrasting patterns emerge in hepatobiliary and gastrointestinal malignancies (biliary tract cancer, colorectal carcinoma, hepatocellular carcinoma), where high-risk PANRS groups correlate with poor prognosis, while low-risk patients demonstrate increased chemosensitivity and potential immunotherapy benefits [19, 64, 65]. Similarly, in breast cancer, He et al. download breast cancer data and GSE176078 single-cell sequencing dataset, construct prognostic models, COX and LASSO regression to establish PANoptosis-based molecular classification, and show low-risk clusters exhibit favorable prognosis and higher degree of microsatellite instability (MSI), suggesting immunotherapy responsiveness [66]. Transcriptomic analyses from 32 different cancer types obtaining from TCGA also reveal tissue-specific prognostic associations of PANoptosis effectors. Adverse outcomes correlate with ZBP1/ADAR/caspase-2,3,4,8/GSDMD overexpression in low grade gliomas and renal carcinomas, while AIM2/caspase-3,4/TNFRSF10 upregulation associates with favorable prognosis in skin cutaneous melanomas [67]. In breast cancer, PANoptosis model based on multi-center cohorts indicates that high-risk patients show higher recurrence and mortality risk and worse prognosis, which was confirmed by immunohistochemistry outcomes of 30 patients [68]. In addition, studies further reveal the predictive role of PANoptosis-related noncoding RNA signatures in cancers. Risk stratification using PANoptosis-related microRNAs (clear cell renal cell carcinoma) or long non-coding RNAs (hepatocellular/thyroid carcinomas) identifies high-risk groups with advanced tumor stages and reduced survival, yet increased responsiveness from immunotherapy and chemotherapy [69–71]. Similar studies have explored this approach in other tumors, including head and neck carcinoma [72, 73], ovarian cancer [74], melanoma [75] lung adenocarcinoma [76, 77]. These studies reveal PANRS’s prognostic and therapeutic implications across cancers.

PANRS not only predict survival and response to anti-tumor therapy in tumor patients, but also identify suitable populations for different therapeutic regimens. In hepatocellular carcinoma, elevated HPAN-index scores (derived from PANoptosis-associated differentially expressed genes) correlate with poorer prognosis but high response to immunotherapy, whereas low-scoring patients show enhanced sensitivity to targeted therapies like sorafenib [78]. Comprehensive analyses in prostate cancer reveal PANRS-associated risk models can predict response to 56 chemotherapeutic agents, with high-risk groups demonstrating lower IC50 values for paclitaxel, sepantronium, and tozasertib, while low-risk groups exhibiting increased sensitivity to oxaliplatin, sorafenib, and irinotecan [79]. Moreover, cutaneous melanoma patients show reversed patterns with low-risk individuals responding better to paclitaxel, dasatinib, imatinib, and lapatinib, while high-risk patients benefiting more from sorafenib [80]. Breast cancer stratification reveals high-risk patients have worse outcomes but respond to chemotherapies like BI-2536 and ispinesib, whereas low-risk patients benefit more from immunotherapy [68]. Gastric cancer analyses demonstrate low PANoptosis scores associate with favorable prognosis and heightened sensitivity to immunotherapy plus multiple chemotherapeutics (paclitaxel, rapamycin, cisplatin), contrasting with high-risk group responsiveness to EGFR/HER2/AKT inhibitors [81], which is further validated by multiple independent cohorts [82]. In addition, through consensus clustering from GSE89749 cohorts and validation with biliary tract cancer cohort in TCGA, PRGs score system classifies patients into cluster A and cluster B. Cluster A remains high level of PANoptosis, better prognosis and more sensitive to docetaxel, while cluster B remains low level of PANoptosis, shorter overall survival and more beneficial from immunotherapy [65]. Pancreatic cancer stratification via PRG expression identifies two subgroups: PANcluster A with poorer prognosis and more sensitivity to erlotinib, selumetinib and trametinib, whereas patients in PANcluster B are highly sensitive to irinotecan, oxaliplatin and sorafenib [83]. These findings collectively establish PANRS as critical biomarkers for guiding personalized therapeutic strategies, enabling optimized selection of immunotherapy, chemotherapy, and targeted agents based on individual molecular profiles.

### The interplay of PANoptosis between cancer cells and TIME

**Fig. 2:**
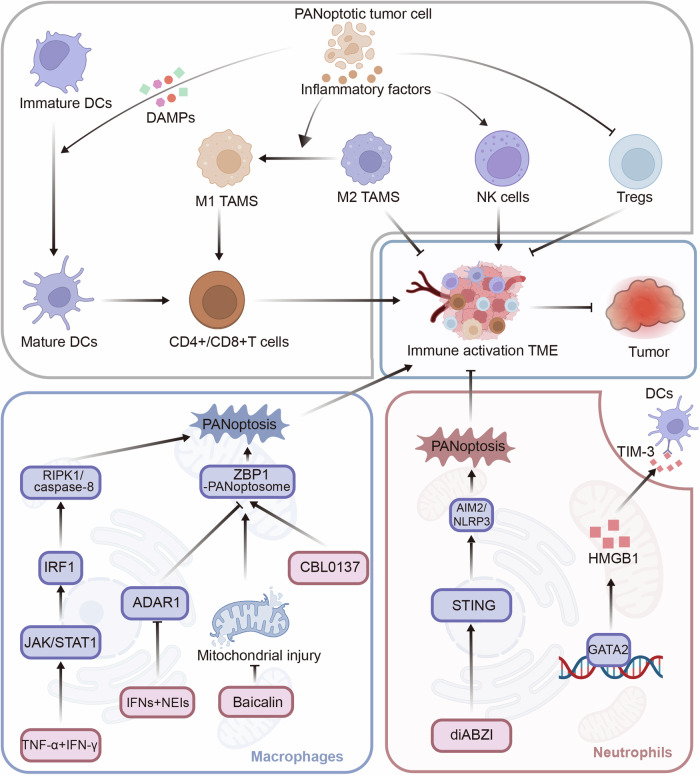
The interplay of PANoptosis between cancer cells and TIME. On the one hand, PANoptotic tumor cells release inflammatory factors or DAMPs, which enhance DCs maturation, M2-to-M1 macrophage repolarization, CD4^+^/CD8^+^ T cell infiltration and activate NK cells, while deplete Tregs to orchestrate immune activation and subsequently suppress tumor development. On the other hand, macrophages are induced to PANoptosis via TNF-α + IFN-γ—JAK/STAT1—IRF1—RIPK1/caspase-8 axis, IFNs+NEIs—ADAR1—ZBP1 axis, or baicalin—mitochondrial injury—ZBP1 axis to enhance inflammatory responses and immune activation (CBL0137 is the agonist of ZBP1), while neutrophils are induced to PANoptosis via diABZI—STING—AIM2/NLRP3 axis and the interaction of GATA2—HMGB1—TIM-3 axis with DCs to inhibit immune activation

TIME play a crucial role in immune surveillance and immune clearance by inducing tumor cells death, but tumor cells develop immune tolerance mechanisms to evade these surveillance systems [84]. The distinct roles of apoptosis, pyroptosis, and necroptosis in modulating TIME and influencing therapeutic outcomes in cancer have been highlighted (Table 3). PANoptosis, a specific form of inflammatory cell death, is considered as an immunogenic cell death accompanied by the release of damage-associated molecular patterns (DAMPs), such as calreticulin (CRT), high mobility group protein B1 (HMGB1), ATP and so on [23]. Thus PANoptosis not only induces cancer cell death directly, but also orchestrate immune activation by recruiting antigen-presenting cells, stimulating cytokine storm, and reprogramming immunosuppressive cellular networks within the TIME (Fig. 2). Mechanistic studies demonstrate the immune stimulatory potential of PANoptosis. Wang et al. reveal that oncolytic virus (OV)-based therapies trigger PANoptosis, which amplifies inflammatory responses through immune activation associated dendritic cell (DCs) maturation, CD8^+^ T cell infiltration, and M2-to-M1 macrophage repolarization, while markedly depleting regulatory T cells (Tregs) [85]. Parallel investigations in hepatocellular carcinoma models show DNASE1L3-mediated PANoptosis enhances anti-tumor immunity. High DNASE1L3 expression correlates with elevated inflammatory mediators and increased CD4^+^/CD8^+^ T cells, natural killer (NK) cells, and macrophages compared with low-expression group [86]. Furthermore, PANoptosis induction in breast cancer and glioblastoma models consistently reduces immunosuppressive tumor-associated macrophages and Tregs while expands mature DCs, memory T cells, and tumor-infiltrating CD8^+^ T cells [87, 88]. These findings collectively demonstrate that PANoptosis is a critical driver of anti-tumor immunity by coordinating immune activation and immunosuppressive cell depletion.

**Table 3:** The interplay of inflammatory PCD with tumor immune microenvironment

Type of PCD	PCD inducer	Effects on TIME	Result	Type of cancer	References
Apoptosis	Mitochondrial outer membrane permeabilisation	Activate macrophages and increases infiltration of CD8^+^ T cells.	Anti-tumor	Colorectal cancer	[158]
	BCL2 inhibition	Promote antigen presentation and activation of DC cells.	Anti-tumor	Lung cancer, Fibrosarcomas	[159]
	Anthracycline/CRT translocation	Recruit DC cells and promote anti-tumor immunity.	Anti-tumor	Colorectal cancer	[160]
	Photodynamic therapy (PDT)	Promote maturation and stimulation of DC cells.	Anti-tumor	Bladder carcinoma	[161]
Necroptosis	FADD—RIPK3	Promote maturation of DCs and cross-priming of T cells through release of DAMPs.	Anti-tumor	Colorectal cancer	[162]
	RIPK3	Promote maturation of DCs and release of IL-1a, IL-12.	Anti-tumor	Cervical cancer	[163]
	Radiotherapy—ZBP1—MLKL	Activate cGAS—STING signaling and increase infiltration and cross-priming of IFN-γ^+^ TNF-α^+^ CD8^+^ T cells.	Anti-tumor	Melanoma, Colorectal cancer, Breast cancer	[139]
	RIPK1/RIPK3	Promote infiltration of BATF3^+^ cDC1 cells and CD8^+^ T cells	Anti-tumor	Melanoma	[164]
	Chemotherapy - RIPK1/RIPK3	Upregulate expression of CXCL1, increasing infiltration of immunosuppressive macrophages.	Pro-tumor	Pancreatic ductal adenocarcinoma	[138]
Pyroptosis	NLRC4 inflammasome	Induce colonic inflammation to promote tumorigenesis.	Pro-tumor	Colorectal cancer	[165]
	GSDME	Enhance phagocytosis of tumor-associated macrophages, increase infiltration of NK and CD8^+^T cells.	Anti-tumor	Breast cancer	[166]
	Phe—BF3— GSDMA3	Infiltration of CD4^+^ and CD8^+^ T cells are increased, and the percentage of CD4^+^ Treg cells is decreased.	Anti-tumor	Breast cancer	[167]
	BRAF inhibitor + MEI inhibitor	induce release of HMGB1, increasing infiltration of CD8^+^, CD4^+^ T cells.	Anti-tumor	Melanoma	[168]

Studies also highlight the critical roles of PANRS in delineating TME traits across various cancers. In thyroid cancer, pathway enrichment analysis demonstrates that PRGs predominantly associate with immunomodulatory processes and TNF signaling pathway, suggesting their potential involvement in macrophage-mediated immune dysregulation and lymphocyte activation [89]. Distinct immune landscape patterns emerge when analyzing PRG-based risk stratification in colorectal and hepatocellular carcinomas. High-risk scores are positively correlated with pro-tumor immune components (M0/M2 macrophages, activated mast cells, and neutrophils), while negatively associated with anti-tumor immune elements (activated DCs, M1 macrophages, resting mast cells, and memory CD4^+^/CD8^+^ T cells) [18, 19]. Notably, microenvironmental polarization shows tumor-type specificity. Aggressive glioma, clear cell renal cell carcinoma, prostate adenocarcinoma and hepatocellular carcinoma display enhanced anti-tumor immunity in high-risk groups, evidenced by elevated immune cell infiltration, upregulated checkpoint molecules, and low tumor immune dysfunction and low exclusion scores [20, 64, 71, 87]. Conversely, immunosuppressive microenvironments dominate high-risk kidney renal clear cell carcinoma and breast cancer cases, characterized by reduced cytotoxic lymphocyte infiltration (CD4^+^ T/NK cells), diminished checkpoint expression, and increased M2 macrophages/Treg populations [66, 90]. In addition, pan-cancer pathway analysis further establishes significant associations between PANoptosis signatures and key immune regulatory mechanisms, including inflammatory response pathways, immune cell infiltration patterns, and immune-related gene networks [82, 91]. These different immune regulatory profiles may explain the different immunotherapy response among patients with distinct PANRS.

Immune cells not only express PANoptosis-related genes but also undergo PANoptosis, significantly influencing tumor immune dynamics (Fig. 2). Experimental models reveal distinct activation mechanisms in macrophages and neutrophils. In macrophage, adenosine deaminase acting on RNA 1 (ADAR1), an RNA editor to maintain homeostasis of post-transcriptional RNA base modification, suppresses PANoptosis by interacting with the Zα2 domain of ZBP1 to limit ZBP1 and RIPK3 interactions, while combining interferons (IFNs) and nuclear export inhibitors (NEIs: KPT-330 or LMB) treatment induces PANoptosis in ZBP1-dependent manner [92]. TNF-α/IFN-γ co-stimulation also trigger caspase-8/FADD-mediated PANoptosis through JAK/STAT1/IRF1 axis activation and nitric oxide production [15]. Pharmacological modulation studies highlight therapeutic potential. Baicalin (a scutellaria baicalensis flavonoid) suppresses PANoptosis, whereas curaxin CBL0137 promotes ZBP1-PANoptosome assembly in macrophages, respectively inhibiting or inducing inflammatory responses, which may activate the TIME[93, 94]. In addition, The DNA damage adapter stimulator of interferon genes (STING) agonist diamidobenzimidazole (diABZI) induces neutrophilic inflammation and PANoptosis via Toll-like receptor 9 (TLR9) upregulation coupled with NLRP3/AIM2 inflammasome activation [95]. Tumor-associated neutrophils (TANs) display elevated PANoptosis gene expression and play pro-tumor activity with DCs via GATA-binding protein 2 (GATA2)-HMGB1-TIM-3 pathway, thereby hindering anti-tumor immunity and facilitating immune evasion of non-small cell lung cancer cells [96]. Relevant sequencing analyses also reveal cell-type specific PANoptosis patterns. Yi et al. find CD8^+^ T cells, B cells, and DCs show higher PANoptosis signature scores compared with other immune populations in prostate adenocarcinoma, indicating that these cells have more severe PANoptosis [20]. Similarly, Dai et al. show that high PANscore clustered relatively in B cells, and low PANscore in CD16^+^ and CD14^+^ monocytes and megakaryocyte progenitors based on single-cell sequencing data [97]. Hepatocellular carcinoma studies further demonstrate differential PRG expression across immune cells, such as upregulated CRADD in tumor-infiltrating Tregs versus elevated TNF in cytotoxic FGFBP2^+^ T cells [27–29]. These studies provided critical insights to understand the mechanism of PANoptosis in cancers and tumor microenvironment. Investigating the distinct types of immune cells and their ability to mediate anti-tumor or pro-tumor upon cellular context is crucial, particularly their interaction with PANoptosis, which inspired us the therapeutic intervention to regulate the interaction between PANoptosis and TIME.

## Therapeutic implications for targeting PANoptosis in cancer

Targeting PCD regulators through genetic or pharmacological interventions has gained significant progress in oncology research. Various compounds or inhibitors targeting the sensor or execution proteins of PCD, such as caspases, NLRP3 or RIPK1/3, have been proposed to regulate cell death [98, 99]. Notably, recent advances demonstrate that multiple anticancer agents and nanoparticle-based systems can effectively trigger PANoptosis signaling pathways to offer novel therapeutic strategies for tumor treatment [88, 100]. Targeting PANoptosis not only synergistically activates multiple cell death pathways, overcome therapy resistance, but also promotes anti-tumor immune responses by recruiting antigen-presenting cells, stimulating cytokine storm, and reprogramming immunosuppressive cellular networks within the TIME [85, 101]. PANoptosis related sensors, PANoptosome components, or upstream regulatory networks present promising candidates for developing combination therapies to overcome therapeutic challenges in cancer management.

### Directly modulating PANoptosome components

ZBP1-mediated PANoptosis plays an important role in the anti-tumor effects in various tumors. Small-molecule inhibitors, such as necrosulfonamide, targeting ZBP1’s RHIM domain have shown efficacy in reducing intestinal damage in murine models [102]. Depletion of ZBP1 markedly decrease PANoptotic cell death in adrenocortical carcinoma, breast cancer and head and neck squamous cell carcinoma cells, and results in a significant tumorigenesis in colorectal cancer, melanoma and hemophagocytic lymphohistiocytosis mice models [85, 92, 93, 103]. Conversely, ZBP1 agonists, such as CBL0137, are under investigation to enhance tumor cell death in ZBP1-deficient cancers [24]. AIM2 PANoptosome also acts as a tumor suppressor by eliminating transformed cells through PANoptosis in colorectal cancer [104], and the downregulation of AIM2 led to accelerated proliferation of melanoma cells [105]. However, chronic inflammation in the TME often silences AIM2 via promoter hypermethylation, enabling immune evasion. Therapeutic strategies to demethylate AIM2 or deliver AIM2-encoding vectors are being explored to restore its tumor-suppressive function [106]. In addition, the pan-caspase inhibitor emricasan can inhibit the PANoptosis induced by co-treatment of IFN-γ and TNF-α and reduce cell death in DNA mismatch repair deficiency (dMMR) tumor [107]. However, due to its complex mechanism, the role of PANoptosis mediated by caspase-5, caspase-8 and NLRP12 in cancer remains controversial. Yang et al. show that reducing caspase-5 level suppresses the growth, migration, and invasion of clear cell renal cell carcinoma cells [108]. For caspase-8, high expression of caspase-8 in the nucleus of tumor cells promotes tumor progression, while inhibits tumor growth by acting as a downstream of granzymes, specifically inducing GSDME cleavage [109]. For NLRP12, it is shown to suppress colitis-associated colorectal cancer by limiting IL-1β production, while exist pro-tumor effects via NF-κB activation in prostate cancer [110, 111]. These findings highlight the conditional therapeutic value of PANoptosome targeting, emphasizing the need for context-specific modulation strategies in cancer treatment.

### Targeting the regulatory network of PANoptosis

**Fig. 3:**
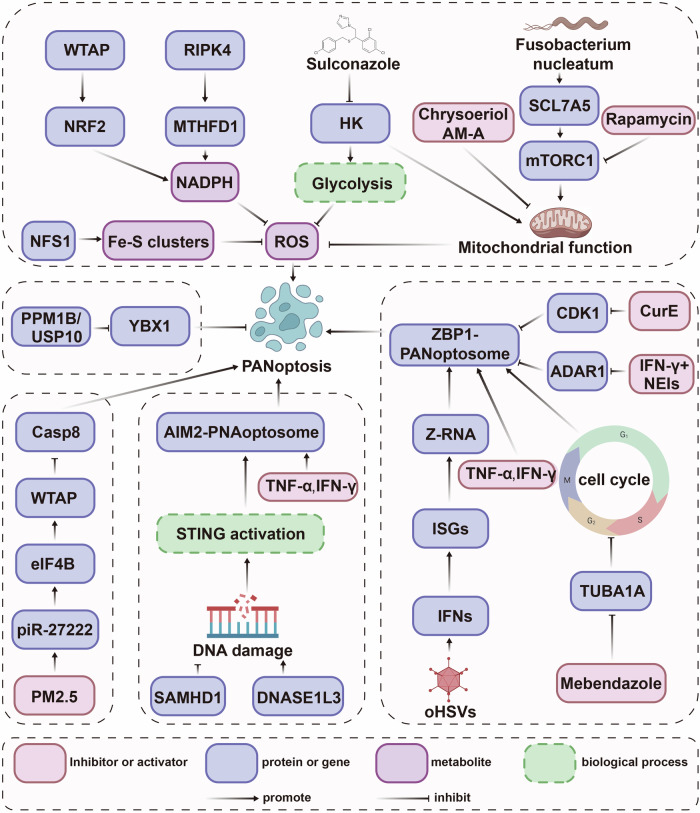
The regulatory network of PANoptosis in cancer cells. In cancer cells, PANoptosis is induced by ROS accumulation, which can be revised by NFS1—Fe-S clusters axis, WTAP—NRF2—NADPH axis, RIPK4—MTHFD1—NADPH axis, sulconazole—HK—glycolysis/mitochondrial function axis, and fusobacterium nucleatum—SLC7A5—mTORC1—mitochondrial function axis (rapamycin is the inhibitor of mTORC1, chrysoeriol and AM-A are natural drug that inhibit the mitochondrial function). Besides, the SAMHD1/DNASE1L3—DNA damage—STING axis and TNF-α + IFN-γ activate AIM2-PNAoptosome, and oHSVs—IFN—ISGs—Z-RNA axis, mebendazole—TUBA1A—cell cycle axis, and TNF-α + IFN-γ activate ZBP1-PNAoptosome to trigger PANoptosis. Moreover, the PANoptosis is inhibited by CurE—CDK1 axis, IFN-γ + NEIs—ADAR1 axis, PM2.5—piR-27222—eIF4B—WTAP axis, and PPM1B/USP10—YBX1 axis

PANoptosis is regulated by multiple upstream signaling pathways under the stimulation of the intracellular and extracellular stimuli (Fig. 3). Targeting these pathways can induce PANoptosis to provide therapeutic strategies for tumor treatment. Metabolic reprogramming, a crucial hallmark of malignant cells, plays a crucial role in regulating PANoptosis [1]. Central to this regulation is ROS accumulation from oxidative stress imbalance, which amplifies PANoptosis through effector protein activation. Antioxidants like N-acetyl-l-cysteine (NAC) effectively neutralize ROS to block this process [13, 17]. Mechanistic studies identify multiple ROS regulatory nodes. Depletion of NFS1 disrupts iron-sulfur (Fe-S) cluster biogenesis, inhibition of m^6^A methyltransferase WTAP reduces the stability of NRF2 mRNA in an m^6^A-dependent manner, and loss of RIPK4 dephosphorylate methylenetetrahydrofolate dehydrogenase 1 (MTHFD1) to inhibit NADPH production, all these result in the increase of intracellular levels of ROS, which subsequently significantly triggers PANoptosis to attenuate CRC cell growth [13, 17, 112]. Consistently, phenyllactic acid enhances prostate cancer cell survival by promoting ATP generation, enhancing NFS1 expression, reducing TNF-α levels, thereby inhibiting PANoptosis, suggesting phenyllactic acid as a novel therapeutic target [113]. Moreover, DNA damage response pathways also show significant effect on PANoptosis. In diffuse large B-cell lymphoma, sterile alpha motif and HD domain-containing protein 1 (SAMHD1) deficiency induces PANoptosis and inhibits tumor growth by inducing DNA damage and promoting the expression of STING, which promotes the formation of caspase-8/RIPK3/ASC complex [114]. Deoxyribonuclease 1 like 3 (DNASE1L3) facilitates double-strand deoxyribonucleic acid (dsDNA) breaks generation and activates AIM2 pathway during sorafenib treatment to trigger PANoptosis and decrease the hepatocellular carcinoma cell survival [86]. In addition, genetic interventions targeting PPM1B/USP10-Y-box binding protein 1 (YBX1) axis or lysophosphatidylcholine acyltransferase 1 (LPCAT1) enhance PANoptosis-related protein activation, effectively suppressing gastric and hepatocellular carcinoma progression [16, 64].

Pharmacological agents exhibit PANoptosis-modulating capacities through distinct pathways. The antifungal agent sulconazole promotes PANoptosis and inhibits the proliferation and migration of esophageal cancer by triggering mitochondrial oxidative stress and inhibiting glycolysis via downregulation of hexokinases (HKs) [115]. Chrysoeriol, a natural flavonoid, shows significant suppressive effects on melanoma progression via inducing PANoptosis by altering mitochondrial membrane potential, increasing ROS production and up-regulating molecules linked to PANoptosis [105]. Atramacronoid A (AM-A), a unique natural sesquiterpene lactone, reduces the proliferation of breast cancer by activating caspase-3/PARP-GSDMD-MLKL cascades through mitochondrial dysfunction [116]. Moreover, the CDK1 inhibitor CurE induces ZBP1-dependent PANoptosis and suppresses the proliferation of adrenocortical carcinoma cells, and adenosine deaminase acting on RNA 1 (ADAR1) depletion exerts similar effects under the treatment of NEIs plus IFN-γ in colorectal cancer and melanoma models [92, 103]. In addition, mebendazole (MBD), an FDA-approved cost-effective anthelmintic drug, inhibits tubulin α1A (TUBA1A), blocks cell cycle and promotes ZBP-1 mediated PANoptosis, simultaneously inhibits tumorigenesis and invasion of acute myeloid leukemia cells [117]. Notably, microbial interactions further modulate PANoptosis dynamics. Fusobacterium nucleatum infection attenuates radiation-induced PANoptosis in nasopharyngeal carcinoma cells via the SLC7A5/leucine-mTORC1 axis to maintain energy metabolism and ROS reduction. mTORC1 inhibitor rapamycin significantly reverses the effects mediated by Fusobacterium nucleatum [118]. Given the emerging critical roles of microbiota in tumorigenesis and progression, the interplay between other microbial species and PANoptosis-related signaling pathways needs systematic investigation to elucidate their potential impact on tumor intervention.

Cytokine combinations, particularly TNF-α and IFN-γ, demonstrate broad PANoptosis-inducing capacity across 13 human cancer models, where MLH1 deficiency activates the AIM2-ZBP-ASC-RIPK1-RIPK3-caspase8 complex, accompanied by hyperactivation of PANoptosis-related effector proteins [107]. The intratumoral administration of TNF-α and IFN-γ exhibits pan-cancer therapeutic efficacy [119]. Notably,IRF1 orchestrates PANoptosis in both myeloid and epithelial cells to induce cell death, suppressing colorectal tumorigenesis through dual-compartment regulation [26]. The interferon response, induced by HSV-1-based OVs (oHSVs), can facilitate the accumulation of endogenous Z-RNA and subsequent activation of ZBP1-mediated PANoptosis to achieve an anti-tumor effect [85]. Nuclear export inhibitors synergize with IFN-γ to enhance melanoma cell death via ZBP1 upregulation and PANoptotic marker activation [67]. In addition, Environmental carcinogenesis studies reveal PM2.5 pollution impairs PANoptosis and promotes lung cancer through piR-27222-mediated eIF4B/WTAP/m6A axis. This effects notably reversed by piR-27222 knockdown [120]. Collectively, these findings demonstrate that targeting the regulatory network of PANoptosis represents a promising therapeutic strategy across multiple cancer types.

### Targeting PANoptosis via nanomedicine

In nanomedicine research, engineered nanoparticles including extracellular vesicles, liposome carriers, dendrimer structures, and polymer matrices serve as versatile platforms for precise delivery of chemotherapeutic agents, genetic materials, and bioactive compounds [121, 122]. The integration of nanotechnology in cancer treatments has emerged as a transformative approach that effectively addresses key challenges in conventional drug delivery systems. These nanoscale innovations significantly enhance tumor-targeting precision, improve water solubility of therapeutic reagents and optimize bioavailability, while minimize systemic toxicity [123–125]. Notably, recent advances demonstrate their capacity to induce PANoptosis and promote the therapeutic effect of cancers.

Innovative delivery platforms induce PANoptosis for immune activation and anti-tumor therapy. Zhou et al. develop nano/genetically engineered extracellular vesicles that induce tumor highly immunogenic PANoptosis, characterized by DAMPs release (CRT exposure, HMGB1/ATP secretion) promoting DC maturation and macrophage polarization. This initiates cGAS-STING-mediated T cell responses, effectively overcoming tumor immunosuppression [126]. Similarly, mesoporous piezoelectric SrTiO3 nanoparticles (MeST NPs), combined with bioactive glasses scaffolds, generate ROS to disrupt cellular integrity, inducing PANoptosis to drive immune activation through DAMP release and enhanced T cell infiltration, which significantly suppress osteosarcoma growth [22]. Targeted co-delivery systems further demonstrate synergistic potential. pH-sensitive alginate-cholesterol-folic acid nanoparticles (FCA NPs) co-deliver a combination of metformin (MET) and doxorubicin (DOX), thus induce PANoptosis and block progression of melanoma cell through concurrent pyroptotic, apoptotic, and necroptotic pathways [100]. Moreover, sonodynamic therapy (SDT) utilizes sonosensitizers and oxygen to generate ROS and induce oxidative damage of tumor cells, but the efficiency of sonosensitizers in generating reactive ROS is often limited by rapid electron-hole recombination [127, 128]. Bi-based nanocomposites, such as BiF3@BiOI@Pt-PVP (BBP) and Bi@Bi2O3-Pt-PEG (BBOP), address ROS generation limitations to enhance SDT efficacy by prolonging electron-hole separation, amplifying mitochondrial damage and glutathione depletion to induce PANoptosis, thereby synergistically inhibit breast tumor growth and lung metastasis [129, 130]. In addition, emerging nanoplatforms combine multiple therapeutic modalities, such as covalent organic frameworks (COFs) that are porous crystals of high potential in biomedical application [131]. The COF-based PorSe-CuPt@CBL, preloaded with ZBP1 agonist CBL0137 under radiotherapy treatment, generates substantial amounts of ROS, which effectively trigger ZBP1-mediated PANoptosis and potentiate breast cancer treatment [88]. These observations indicate that targeting PANoptosis cell death via nanoengineering may offer unique opportunities to boost anti-tumor therapy.

## Combination therapies involving PANoptosis modulation

Although the existing therapeutic regimens, including radiotherapy, chemotherapy, targeted agents and immunotherapy, have obtained curative effects for cancers, the associated side effects and hyposensitivity are the main factors contributing to treatment failure for refractory patients [132]. The studies on the role and mechanism of PANoptosis provide a promising strategy to enhance the efficacy of existing cancer therapies (Table 4).

**Table 4:** Combination therapies involving PANoptosis modulation

PANoptosis inducer	Related pathway	Enhanced sensitivity	Type of cancer	References
NFS1 suppression	Inhibit Fe-S cluster biogenesis—ROS accumulation	Oxaliplatin	Colorectal cancer	[13]
WTAP suppression	Decrease NRF2 m^6^A—ROS accumulation	Oxaliplatin	Colorectal cancer	[17]
YBX1 suppression	Activate PANoptosis executors	Oxaliplatin	Gastric cancer	[16]
RIPK4 loss	Dephosphorylate MTHFD1—NADPH reduction—ROS accumulation	Oxaliplatin	Gastric cancer	[112]
FADD knockdown	Inhibit MAPK and mTOR signal	5-fluorouracil	Head and neck squamous cell carcinoma	[72]
CurE	Inhibit CDK1—trigger ZBP1-PANoptosome	Mitotane	Adrenocortical carcinoma	[103]
Sulconazole	Trigger oxidative stress and inhibit glycolysis	Radiotherapy	Esophageal cancer	[115]
Mitochondria-targeted antibiotics or leucine dietary restriction	Reduce Fusobacterium nucleatum —ROS accumulation	Radiotherapy	Nasopharyngeal carcinoma	[118]
PorSe-CuPt@CBL	ROS accumulation—trigger ZBP1-PANoptosome	Radiotherapy	Breast cancer	[88]
BiF3@BiOI@Pt-PV/ Bi@Bi2O3-Pt-PEG	ROS accumulation	Sonodynamic therapy	Breast cancer	[129, 130],
DMXAA	Activate cGAS—STING signal	PD-L1 blockade	Diffuse large B-cell lymphoma tumor	[114]
DNASE1L3 overexpression	DNA damage—trigger AIM2-PANoptosome	Sorafenib/ PD-1 blockade	Hepatocellular carcinoma	[86]
Cinobufagin	Mitochondrial damage and oxidative stress	PD-1 blockade	Glioblastoma	[87]
VSV-S	ROS accumulation	PD-1 blockade	Head and neck squamous cell carcinoma	[137]
oHSVs	Up-regulate interferon-stimulated genes—trigger ZBP1-PANoptosome	PD-L1/CTLA-4 blockade	Breast cancer	[85]

### Enhancing the efficacy of tumor chemoradiotherapy

Emerging evidence highlights PANoptosis activation as a strategic enhancer of chemoradiotherapy efficacy. Cytotoxic drugs are conventional first-line chemotherapy drugs for various cancers. In gastrointestinal cancers, NFS1, WTAP or YBX1 suppression significantly improves oxaliplatin efficacy by inducing PANoptosis, thereby overcoming chemoresistance in colorectal and gastric cancer models [13, 16, 17]. Similarly, FADD knockdown in head and neck squamous cell carcinoma not only inhibits tumor proliferation but also improves 5-fluorouracil responsiveness [72]. Mitotane is the only first-line treatment for adrenocortical carcinoma. The therapeutic effect of a combination of CDK1 inhibitor CurE and mitotane in adrenocortical carcinoma cells achieves superior therapeutic outcomes through PANoptosis activation compared to monotherapies [103]. Radiotherapy optimization studies reveal microenvironment-dependent mechanisms. Sulconazole is effective in inhibiting the growth of esophageal cancer cells, and the combination of low dose sulconazole and radiotherapy promotes PANoptosis through oxidative stress activation and glycolytic pathway inhibition to increase radiosensitivity [115]. In radioresistant nasopharyngeal carcinomas, microbiota modulation through mitochondrial-targeted antibiotics or leucine-restricted diets restores radiation sensitivity by reducing Fusobacterium nucleatum-mediated PANoptosis suppression [118]. In addition, nanotechnology applications show particular promise, with PorSe-CuPt@CBL nanoparticles effectively triggering PANoptosis to boost radiotherapy effectiveness in breast cancer [88]. Notably, ZBP1-mediated PANoptosis contributes to the toxic side effects in healthy tissues of colorectal cancer patients under DNA-damage therapies, suggesting ZBP1 can be a promising therapeutic target to alleviate chemotherapy-related side effects [133]. Therefore, combining PANoptosis inducers with radiotherapy or chemotherapy demonstrates synergistic anti-tumor effects, highlighting PANoptosis as a pivotal modulator of treatment response and a candidate for precision oncology.

### Enhancing the efficacy of immunotherapy

Anti-tumor immunotherapies have arisen as fresh therapeutic pillars within oncology by countering tumor-induced immunosuppression, but their clinical effectiveness remains limited by acquired resistance and immune evasion mechanisms. Emerging evidence positions PANoptosis induction as a strategic enhancer of immunotherapy efficacy through TME reprogramming [86, 114]. Mechanistic studies demonstrate DNASE1L3-induced PANoptosis augment the activation of anti-tumor immunity within the TME, thereby enhancing the efficacy of sorafenib/programmed death-1 (PD-1) mAb combination therapy in hepatocellular carcinoma [86]. Similarly, STING pathway activation via genetic overexpression or agonist DMXAA promote PANoptosis to enhance programmed death-ligand 1 (PD-L1) blockade efficacy and suppress diffuse large B-cell lymphoma tumor growth [114]. Bioactive compounds like cinobufagin synergize with PD-1 inhibitors in glioblastoma models, achieving survival prolongation via PANoptosis-driven immune activation [87]. In addition, oncolytic viruses (OVs) are increasingly recognized as promising tools for cancer therapy, as they selectively infect and destroy tumor cells while leave healthy cells unharmed [134]. However, the potential of oncolytic virus (OV) for cancer therapy is limited by the efficiency of induced immune response [135, 136]. It shows particular promise when combining with PANoptosis induction. Herpes simplex virus-1 (HSV-1)-based OVs (oHSVs) or SMAC/DIABLO gene-inserted vesicular stomatitis virus (VSV)-based OVSs (VSVs) potentiate anti-tumor immunity by inducing PANoptosis, promoting T cell tumor infiltration and enhancing their cytotoxic capacity. oHSVs or VSV-S in combination with PD-1 blockade, produces a more potent anti-tumor effect than either therapy alone [137]. Innovative combinations using Fusobacterium nucleatum outer membrane vesicles (Fn-OMV) with oHSVs convert immunologically cold tumors to hot phenotypes, amplifying PANoptosis-mediated inflammation to improve PD-L1/CTLA-4 therapy [85]. In addition, nanotechnological advances further expand therapeutic possibilities, with nanocomposites (BBP/BBOP) demonstrating PANoptosis-triggering capacity and immunotherapy enhancement in breast cancer models [129, 130]. These strategies underscore PANoptosis as a key modulator of immune evasion, enabling precision oncology approaches. However, optimizing combinatorial regimens and addressing tumor heterogeneity remain critical for clinical translation.

## Conclusive remarks and future perspectives

The evolving landscape of PANoptosis has established its central role in shaping tumorigenesis, immune evasion, and therapeutic response. By orchestrating apoptosis, pyroptosis and necroptosis through PANoptosome-mediated signaling, this multifaceted cell death pathway not only amplifies immunogenicity but also interacts with the TME to influence cancer progression. In clinical and preclinical models, integrating multi-omics data and machine learning, derived from the expression pattern of PANoptosis-related signatures reveal the great potential in predicting patient prognosis, discerning tumor microenvironment traits, and predicting response to existing anti-tumor therapy. However, due to the complex mechanism and the context heterogeneity of PANoptosis activation in cancers, its molecular drivers and downstream effects exhibit tumor-type specificity. For instance, the high-score group of PANoptosis signature correlate with better survival outcomes and better immunotherapeutic responses in prostate cancer, while exhibit poor prognosis, lower response to chemotherapeutic agents and immunotherapy in biliary tract cancer, colorectal carcinoma and hepatocellular carcinoma [19, 20, 64, 65]. This heterogeneity may arise from tissue-specific signaling contexts or differences in microbiota composition. This underscores the need for systematic single-cell and spatial multi-omics studies to dissect PANoptosis signatures across tumor subtypes and microenvironmental niches. In addition, patients with distinct PANRS profiles exhibit differential therapeutic sensitivities to various treatments, including chemotherapy, immunotherapy, or specific chemotherapeutic agents. The development of specific biomarkers is required to guide personalized therapeutic strategies for different tumor patients. However, most PANRS models are rarely validated by human cohort data, their predictive power must be strictly tested in large, multi-cohort studies, and need further validation by preclinical and clinical studies.

Studies have provided critical insights to understand the mechanism of PANoptosis in bridging direct cancer cell elimination with TIME, in which PANoptotic tumor cells can enhance anti-tumor immunity by promoting DC maturation, T-cell infiltration, or M1 macrophage polarization [86–88]. However, there exhibits contrasting effects of PANoptosis across different tumor types, which may be related to the activation differences of the TIME, tumor-tissue heterogeneity or complex molecular mechanisms. PANoptosis signatures correlate with immune activation in glioma or hepatocellular carcinoma but associate with immunosuppression in breast and kidney cancer [64, 66, 71, 90]. Moreover, immune cells themselves exhibit PANoptosis plasticity. For instance, tumor-associated neutrophils upregulate PANoptosis to promote immune evasion in non-small cell lung cancer, while ZBP1-dependent PANoptosis in macrophages amplifies anti-tumor responses [93, 96]. Additionally, RIPK1/RIPK3 mediated necroptosis upregulates the expression of CXCL1, increasing infiltration of immunosuppressive macrophages, resulting in the pro-tumoral role [138], while ZBP1-MLKL necroptotic cascade induces cytoplasmic DNA accumulation, activates cGAS—STING signaling and increases infiltration of IFN-γ^+^ TNF-α^+^ CD8^+^ T cells, mediating the antitumor immunity [139]. These findings underscore the dual role of PANoptosis in immune TME regulation and even tumor treatment, highlighting the necessity of context-specific therapeutic strategies. Moreover, the dynamic interplay between PANoptosis and other immune cells or tumor stromal cells in TME, such as cancer-associated fibroblasts, myeloid progenitors, needs detailed characterization. Moreover, continuous studies tracking PANoptosis-induced DAMP release and its systemic immune consequences will be essential to avoid immune-related adverse events. Overall, further studies of the relationship between TME and PANoptosis could help to gain a deeper understanding of the tumor immune escape mechanism and provide new ideas for the development of tumor immunotherapy strategies.

PANoptosis emerges as a therapeutic vulnerability in cancers and targeting PANoptosis-related pathways offers unique opportunities to boost anti-tumor efficacy. Many activators or inhibitors associated with PANoptosis have been discovered and verified to enhance tumor cell death, such as ZBP1 agonists CBL0137 [24]. However, the role of PANoptosis has mainly been assessed in vitro and in murine models. Due to the limitations in uniformly controlling the TME, the significant heterogeneity in the activation degree of immune cells among different tumor types and variability in patient responses with monotherapy, their safety profiles and more optimized combinatorial regimens remain uncertain, which may inadvertently trigger compensatory cell death to raise toxicity. Further investigation and clinical trials are essential to validate the safety and efficacy of PANoptosis targeted treatments. Clinical trials must adopt adaptive designs to accommodate the complexity of PANoptosis biology, including patient stratification based on genetic, epigenetic, and immunophenotypic markers. In addition, other forms of cell death include cuproptosis, disulfidptosis and alkaliptosis have also been documented as emerging PCD pathways [140–142]. The interplay and regulatory mechanism between PANoptosis and other emerging PCD pathways remain poorly understood. With the discovery of novel PANoptosome and mechanisms, the interaction between PANoptosis and ferroptosis or other PCD will be further analyzed. Unraveling these crosstalk mechanisms will be critical to avoiding unexpected consequences of PANoptosis modulation. Equally pressing is the development of delivery systems to selectively target PANoptosis components in the TME. Nanotechnology-based platforms, such as nanoparticles co-loaded with PANoptosis inducers and immune stimulants, hold translational potential but require optimization for scalability and applicability.

In conclusion, PANoptosis represents a paradigm-shifting concept. The therapeutic implications of PANoptosis in cancer are still emerging, and there is potential for the development of new treatments that target this pathway and improve conventional therapies. Integrating PANoptosis-targeted therapy with existing modalities is likely to pave the way for personalized and precise approaches to cancer treatment in the future.
